# Data Fit for Health Equity: Learning Health Systems, AI, and the STANDING Together Recommendations

**DOI:** 10.1002/lrh2.70053

**Published:** 2025-12-05

**Authors:** Elinor Laws, Neil Cockburn

**Affiliations:** ^1^ Department of Applied Health Sciences University of Birmingham Birmingham UK; ^2^ Warwick Medical School University of Warwick Warwick UK

**Keywords:** artificial intelligence, clinical decision support, health data, health equity, learning health systems

## Abstract

**Introduction:**

Artificial Intelligence (AI) tools may deliver significant improvements in healthcare and Learning Health Systems are well positioned to benefit. However, during the adoption of AI, Learning Health Systems should consider the potential for AI to exacerbate health inequity and perpetuate biases that exist in healthcare and its associated data.

**Methods:**

The STANDING Together recommendations provide a method to identify and report potential bias during the curation of datasets for AI and the development of AI from those datasets. The recommendations could form a key learning cycle within a Learning Health System ensuring transparent reporting of healthcare data use and the implementation of AI healthcare technologies that promote health equity.

**Results and Conclusions:**

Learning Health Systems are well placed to adopt the STANDING Together best practice recommendations for using healthcare data as they are likely to have both the capabilities to implement the recommendations and the strategic goals that will realize the value of health data and AI that promotes health equity. The STANDING Together recommendations are available from www.datadiversity.org.

## 1 Background

Artificial intelligence (AI) holds great promise for addressing some of healthcare's most challenging issues and is increasingly incorporated into Learning Health Systems (LHS) for its powerful predictive and administrative abilities. However, a growing body of literature demonstrates the risk of generating and amplifying bias where AI health technologies are implemented and responsible development is overlooked. In one example, a chest X‐ray image classifier was found to be under‐diagnosing disease in groups already experiencing health inequality. Elsewhere, a tool for healthcare resource allocation systematically underestimated the needs of people who are Black [1, 2]. In both cases, bias present within health data impacted the performance of the AI healthcare technologies, which then led to the exacerbation of existing health disparities [3, 4].

LHS will need to develop and apply AI tools to improve care, so consideration of AI bias will be crucial for responsible innovation [5]. The development of AI healthcare technologies is complex and ranges from problem definition to data collection, outcome definition, model development, and implementation [6]. Within every step of AI development, decisions made by humans can generate biases that could exacerbate health disparities. For example, a developer may choose a particular health condition to focus on due to personal interest, and certain clinical outcomes may be prioritised due to individual clinician experience or preference.

Issues of bias also extend beyond AI developers' decisions around data, as the data themselves reflect widespread social biases. At the local level, systemic issues with healthcare access result in certain groups being under‐represented or missing from so‐called real‐world datasets [7]. At the national level, demographic categories may be inconsistently reported and may capture individual identities coarsely, limiting the ability to determine representation of groups. At the international level, healthcare data availability tends to be confined to a small number of high‐income countries as comprehensive healthcare data curation efforts are resource intensive [8, 9, 10]. And finally, it can be challenging to determine which features of data are driving biased AI outputs. AI can infer demographic attributes like race or ethnicity from unrelated health data even when not explicitly trained to do so, and risks encoding social biases into the AI model [11].

The limited availability of well‐reported, inclusive datasets from diverse settings means that AI technologies risk performing well for only select groups that are unlikely to be those most in need [12]. Many LHS curate datasets and develop AI tools explicitly aiming to address inequities, but part of the AI bias problem stems from a lack of transparency relating to dataset use during AI development. While it is an impossible task to remove all bias within healthcare data, LHS could be at the forefront of improvement by encouraging a healthcare data culture that champions transparency.

To support the development of this culture shift towards transparent healthcare data reporting practices, an international initiative called the Standards for Data Diversity, Inclusivity, and Generalisability (STANDING Together) program was undertaken [3, 4]. This commentary proposes the application of the STANDING Together recommendations to the context of LHS, aiming to stimulate further conversations and innovations in the equitable use of data and AI within LHS.

## 2 Methods

The STANDING Together recommendations advocate for more transparent reporting of dataset limitations and biases, and proactive inquiry of how bias could be introduced during AI development. International experts were convened into a working group with representation from different sectors including patients, computer scientists, healthcare professionals, regulation and policy experts, and academics. The scope and content of the project were guided by a patient and public involvement and engagement committee who drew on their lived experiences of health inequality, providing operational and strategic steer on how the project was conducted.

A literature review of existing dataset documentation guidelines informed initial drafting of the STANDING Together recommendations [4]. Using Delphi study methods, four rounds of voting were completed involving over 350 people from 58 different countries, which culminated in an in‐person consensus meeting to reach a final set of recommendations. The methods relating to the development of the draft recommendations and the conduct of the Delphi study have been published separately [4, 13].

## 3 The Scope of the Recommendations

The STANDING Together recommendations are split into two distinct parts: the first part is intended for those who curate healthcare datasets, and the second part is intended for those who use and analyze healthcare datasets. Developed in the context of data for AI, the recommendations are deliberately principle based to ensure relevance to any setting, and they should be contextualized to address specific issues relevant to the setting within which they are applied. For example, attributes like race or ethnicity are not explicitly listed within the STANDING Together recommendations as these are highly culture‐specific, and flexibility is necessary for users to define relevant attributes within their setting. Additionally, the recommendations are intended to be applicable to all stakeholders but are deliberately positioned to support medical device regulators. The full publication of the recommendations includes explanatory text that accompanies each item to provide insight into how to operationalize them. There is also acknowledgment that it may not be possible for all requirements to be met, but in keeping with the principle of transparency, reporting of any omissions and accompanying reasoning is encouraged.

The full recommendations are available at www.datadiversity.org.

### 3.1 Part 1: Recommendations for the Documentation of Health Datasets

The recommendations for the documentation of health datasets emphasize the importance of accompanying documentation for all health datasets and include 18 items in total. They begin with prompting a summary of the dataset, how to find and access the dataset, and the reasons why the dataset was created. Next, the recommendations outline the necessity for clear and transparent reporting of where the data are originated from, and whether data were sampled or aggregated from other existing datasets. The recommendations prompt consideration of potential data shifts over time, the demographic groups present within the dataset, and any potential sources of bias. Finally, there are items relating to ethics, governance, patient and public participation, and bias and impact assessments.

### 3.2 Part 2: Recommendations for Use of Health Datasets

The recommendations for the use of health datasets outline the importance of documenting the decisions made during the development of an AI technology using healthcare datasets. The purpose of this part of the STANDING Together recommendations is to prompt consideration that decisions made during the AI development process can introduce bias. Bias can be introduced as a result of the technology itself, but there may also be existing groups that experience health disparities who may be at greater risk of experiencing bias from AI health technologies. These groups are referred to within the recommendations as “contextualized groups of interest” and are defined as “groups of individuals with shared relevant attributes that have known or suspected associations with disparate health outcomes related to the intended use of an AI health technology.” The recommendations acknowledge that disparate health outcomes for these groups can arise from a variety of factors including social or structural determinants of health, biological factors, or implementation of technology (including the AI technology itself). It is essential that AI healthcare technologies are developed using datasets that represent the intended use population, with deliberate consideration of contextualized groups of interest.

## 4 STANDING Together in the LHS

**FIGURE 1:**
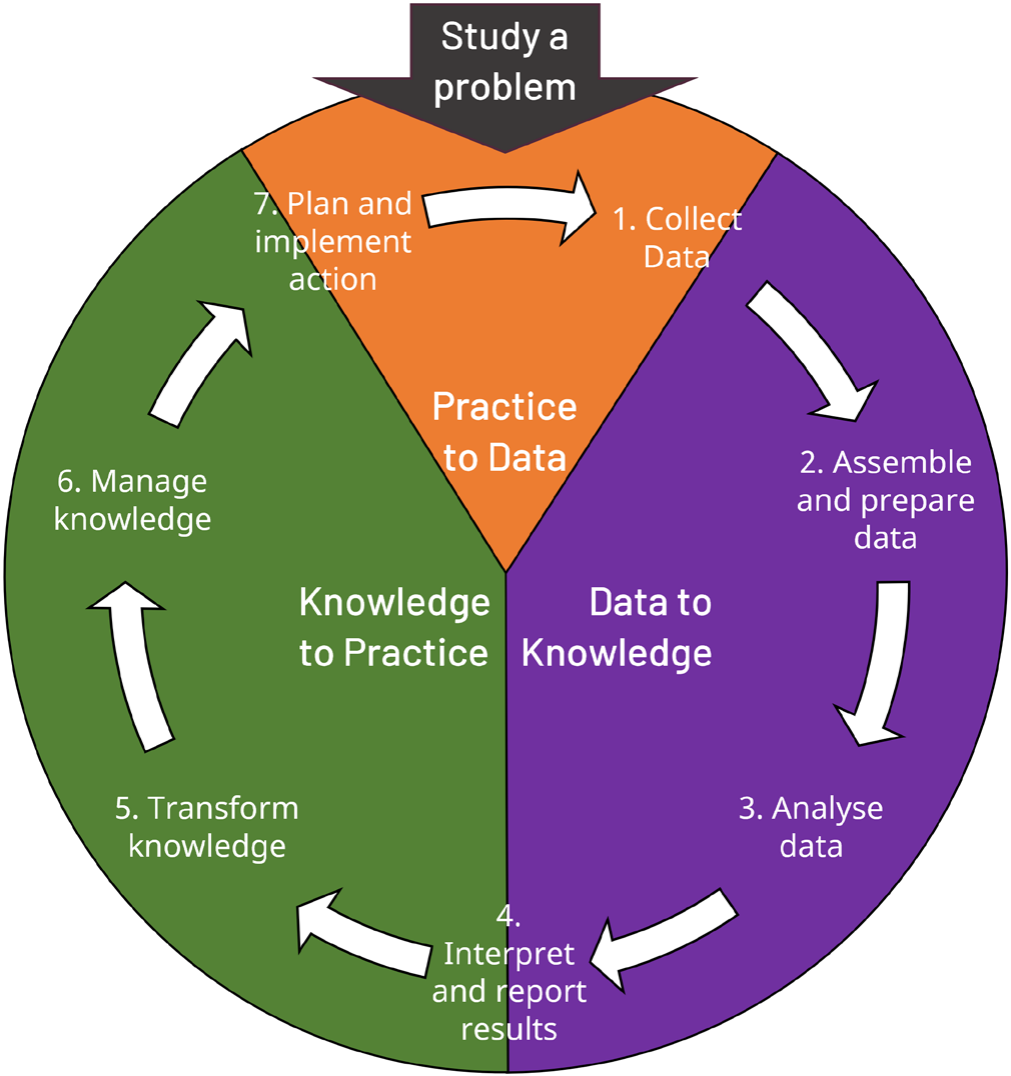
A Learning Cycle, from data to knowledge to practice. Adapted from Flynn et al. [15]

Many LHS are active users of data, and act as data creators, curators, and consumers throughout the course of learning about and improving health. We suggest that the STANDING Together recommendations are particularly apt for LHS, and should form a key learning cycle in LHS. Learning cycles have been described elsewhere as layered, with micro‐level quality improvement nested inside macro‐level system change, with different resource, time, and complexity implications at respective scales [14]. Figure 1 displays the stages of a learning cycle, and the STANDING Together recommendations can be operationalized at many of these stages. For example, the recommendations could be used to guide minimum reporting of data collection practices within LHS, such as the intended purpose for the data, any significant limitations and omissions, and data governance procedures. This would ensure that anyone using the data could have access to transparently reported information around data collection practices to inform any future decision‐making. Mature LHS could incorporate the recommendations into “business as usual” such as during the data collection process or during AI implementation, tracking representation, errors, and missingness to allow responsible decision making and system change.

**FIGURE 2:**
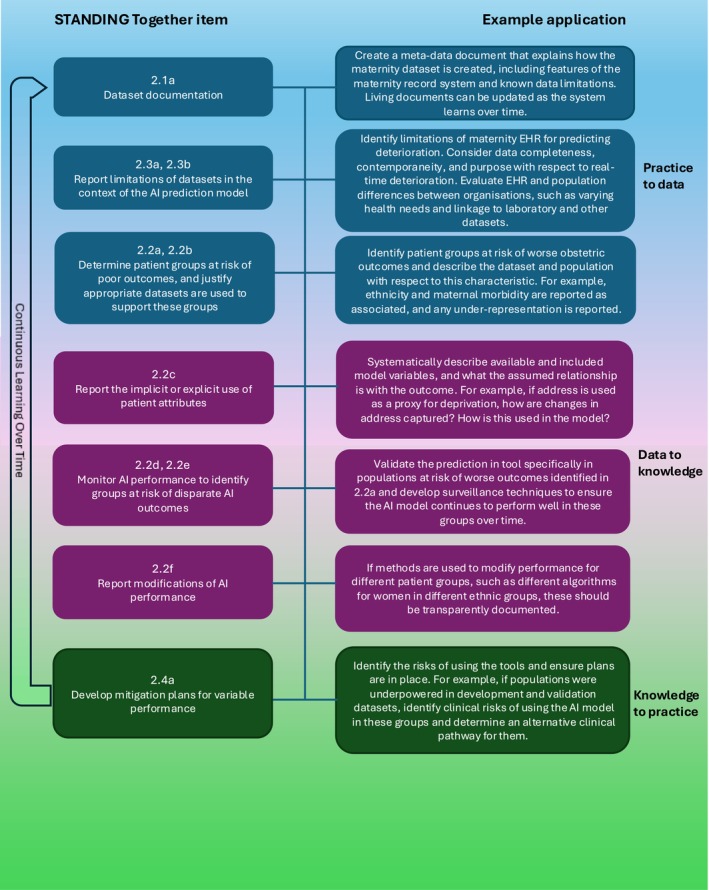
Implementing an AI prediction model to detect deterioration of pregnant women using data from across the electronic health record. EHR, Electronic Health Record.

Figure 2 outlines how the recommendations might be operationalized during the development of an AI technology involving the prediction of maternal outcomes. We consider the case of a LHS developing a tool to detect deterioration in pregnant women, such as planned by the DREaMED study, which specifically aims to address health inequities during pregnancy [16].

Transparent description of the data and its context needs to be available as metadata to any developer of AI tools or other secondary users of data, whether within the LHS or an external dataset contributed to by the LHS, to ensure developers address health inequalities. All LHS could internalize the STANDING Together recommendations as a continuous process of improving, curating, and developing data assets. The recommendations need to be operationalized in the context of the LHS goals and the population it is made up of and serves; LHS should understand the health disparities present within their population and ensure data collected is fit to describe and address these disparities. Implementing the STANDING Together recommendations in full may come at significant resource costs to systems, which may already face financial challenges. However, neglecting to transparently report dataset curation and use may contribute to health disparities and lead to worse health outcomes for those most in need [1, 2].

## 5 Future Directions

STANDING Together are the first recommendations that aim to encourage transparency and proactive evaluation of the impact of health datasets and AI on population groups. To encourage adoption, the STANDING Together recommendations require support from regulators, funders, standards organizations, amongst others. While the recommendations have been acknowledged by different organizations across the world, including the UK's Medicines and Healthcare products Regulatory Agency (MHRA), the Australian Therapeutic Goods Administration (TGA), and UK funder the Medical Research Council (MRC), there is work to be done to improve the dissemination, adoption, and implementation [17, 18, 19]. There is also a lack of tools to support responsible AI development such as documentation standards and operating guidance to accompany the STANDING Together recommendations [4]. At present, the recommendations represent best‐practice guidance, and we expect tools for operationalizing them to be developed and disseminated during adoption.

Successful implementation will require time and resources from developers and implementers of healthcare datasets and AI healthcare technologies, but the end goal must be kept in mind: striving for digital healthcare innovation that is safe, effective, and equitable. These outcomes will likely accrue to patients in the medium to long term after transparent data reporting practices are implemented and risks of AI bias can be mitigated. The recommendations are not designed to be implemented in their entirety in all settings, and their implementation may require variable interpretation by different LHS depending on their context. We encourage users of the STANDING Together recommendations to publish not only documentation itself, but also experiences in implementing the recommendations for the benefit of community learning and development, contributing to a changing culture towards transparent data use.

## Conflicts of Interest

The authors declare no conflicts of interest.

## Data Availability

Data sharing is not applicable to this article as no datasets were generated or analyzed during the current study.
